# Metabolomics and Lipidomics Signatures of Insulin Resistance and Abdominal Fat Depots in People Living with Obesity

**DOI:** 10.3390/metabo12121272

**Published:** 2022-12-15

**Authors:** Yen Chin Koay, Adelle C. F. Coster, Daniel L. Chen, Brad Milner, Amani Batarseh, John F. O’Sullivan, Jerry R. Greenfield, Dorit Samocha-Bonet

**Affiliations:** 1 School of Medical Sciences, Faculty of Medicine and Health, The University of Sydney, Sydney, NSW 2006, Australia; 2 Heart Research Institute, Newtown, NSW 2042, Australia; 3 School of Mathematics and Statistics, UNSW Sydney, Kensington, NSW 2052, Australia; 4 Clinical Diabetes, Appetite and Metabolism Laboratory, Garvan Institute of Medical Research, Darlinghurst, NSW 2010, Australia; 5 Department of Medical Imaging, St Vincent’s Hospital, Darlinghurst, NSW 2010, Australia; 6 BCAL Diagnostics, National Innovation Centre, Eveleigh, NSW 2015, Australia; 7 Department of Cardiology, Royal Prince Alfred Hospital, Camperdown, NSW 2050, Australia; 8 School of Clinical Medicine, St Vincent’s Healthcare Clinical Campus, UNSW Medicine & Health, UNSW Sydney, Darlinghurst, NSW 2010, Australia; 9 Department of Endocrinology and Diabetes Centre, St Vincent’s Hospital, Darlinghurst, NSW 2010, Australia

**Keywords:** obesity phenotypes, tissue insulin resistance, abdominal fat deposition, liver fat, plasma metabolomics, plasma lipidomics

## Abstract

The liver, skeletal muscle, and adipose tissue are major insulin target tissues and key players in glucose homeostasis. We and others have described diverse insulin resistance (IR) phenotypes in people at risk of developing type 2 diabetes. It is postulated that identifying the IR phenotype in a patient may guide the treatment or the prevention strategy for better health outcomes in populations at risk. Here, we performed plasma metabolomics and lipidomics in a cohort of men and women living with obesity not complicated by diabetes (mean [SD] BMI 36.0 [4.5] kg/m^2^, *n* = 62) to identify plasma signatures of metabolites and lipids that align with phenotypes of IR (muscle, liver, or adipose tissue) and abdominal fat depots. We used 2-step hyperinsulinemic-euglycemic clamp with deuterated glucose, oral glucose tolerance test, dual-energy X-ray absorptiometry and abdominal magnetic resonance imaging to assess muscle-, liver- and adipose tissue- IR, beta cell function, body composition, abdominal fat distribution and liver fat, respectively. Spearman’s rank correlation analyses that passed the Benjamini–Hochberg statistical correction revealed that cytidine, gamma-aminobutyric acid, anandamide, and citrate corresponded uniquely with muscle IR, tryptophan, cAMP and phosphocholine corresponded uniquely with liver IR and phenylpyruvate and hydroxy-isocaproic acid corresponded uniquely with adipose tissue IR (*p* < 7.2 × 10^−4^). Plasma cholesteryl sulfate (*p* = 0.00029) and guanidinoacetic acid (*p* = 0.0001) differentiated between visceral and subcutaneous adiposity, while homogentisate correlated uniquely with liver fat (*p* = 0.00035). Our findings may help identify diverse insulin resistance and adiposity phenotypes and enable targeted treatments in people living with obesity.

## 1. Introduction

The liver, skeletal muscle, and adipose tissue are major insulin target tissues and key players in glucose homeostasis [1]. Of these three studied tissues, whole body insulin resistance (IR) is thought to be driven primarily by the liver and skeletal muscle [1]. In the liver, IR manifests with overproduction of glucose during fasting (e.g., overnight), despite the presence of hyperinsulinemia [2], and by impaired suppression of endogenous glucose production (EGP, mainly hepatic) in response to insulin rise following meals [2,3]. In muscle, IR manifests with impaired glucose uptake following meals, resulting in postprandial hyperglycemia [3]. Studies conducted by our group and by others, have classified individuals at risk of developing type 2 diabetes as having predominantly muscle, liver, or combined muscle and liver IR [4,5,6]. Tissue-specific IR may explain sub-optimal response to ‘one-size-fits-all’ nutritional or pharmacological interventions [4,5,7]. Most importantly, phenotyping of individuals with prediabetes based on glycemia, abdominal fat distribution, liver fat, IR, and insulin secretion identified sub-populations at greater risk of developing type 2 diabetes and diabetes complications [8].

Universally, the conventional type 2 diabetes treatment algorithm commences with metformin, which is one of the most commonly prescribed drugs in the US [9]. While the mechanisms by which metformin exerts its glucose lowering effects are still partly uncovered, the liver is thought to be a prime target [7]. If the glycaemia target is not reached, other medications are added in a stepwise fashion until optimal glycemic control is achieved. Newer classes of glucose-lowering medications with cardioprotective benefits include the glucagon-like peptide 1 receptor agonists (GLP-1Ra) and the sodium-glucose co-transporter 2 inhibitors (SGLT2i) [10]. In terms of glucose action, glucose-lowering medications target insulin secretion and muscle, liver, and adipose tissue IR to different degrees [7] and sub-phenotyping individuals at risk of type 2 diabetes may prove advantageous in targeting treatment. Reliable assessment of organ-specific IR requires applying the hyperinsulinemic-euglycemic clamp with infusion of deuterated glucose, the gold-standard for assessing IR. Information gathered using this method includes the glucose infusion rate (GIR) during high dose insulin infusion, reflective of glucose uptake into muscle (normalized to body mass or fat-free mass); basal EGP, and EGP suppression in the presence of low-dose insulin infusion, reflective of hepatic IR in the fasted and fed state, respectively [11]. However, information originating from the clamp is not standardized and its use in the clinical settings is impractical; it is costly, time consuming, and labor intensive.

Mass spectrometry-based metabolomics and lipidomics of plasma may identify new biomarkers and risk scores for early diagnosis, prognosis, prediction, and management of subjects at risk of developing diabetes [12,13,14]. In the present study, we performed plasma metabolomics and lipidomics in a cohort of men and women living with obesity. We performed extensive phenotyping of tissue IR, glucose regulation and body fat distribution and evaluated classical blood markers of metabolic disease. We report the plasma metabolites and lipid correlates of the different phenotypes studied.

## 2. Experimental Design, Materials and Methods 

### 2.1. Study Participants

The study cohort has previously been described in detail [6]. Subjects were studied at the Clinical Research Facility of the Garvan Institute of Medical Research (Sydney) between 2011 and 2013. Inclusion criteria were men and women with age ranging from 18–70 years, living with obesity (BMI > 30.0 kg/m^2^), not complicated by diabetes, renal or liver dysfunction, cardiovascular disease, and cancer. Sixty-four eligible participants were included in the original study [6], of whom sixty-two, who had complete clamp data, were included in this retrospective study. Exclusion criteria were diabetes (diagnosed using the American Diabetes Association guidelines [15]), treatment with medications that affect glucose metabolism (e.g., glucose-lowering medications, glucocorticoids), weight change >5% in the 3 months leading up to the study, renal or liver dysfunction, a history of cardiovascular disease and cancer. The protocol was approved by St Vincent’s Hospital Human Research Ethics Committee (Sydney, HREC/10/SVH/133) and written consent was obtained prior to study commencement.

### 2.2. Measurements of Glucose Regulation

Study participants attended the Clinical Research Facility on 2 separate occasions for OGTT and hyperinsulinemic-euglycemic clamp. Prior to both visits, participants were asked to fast overnight (for 10–12 h), and for 48 h prior, avoid intensive exercise, abstain from alcohol, and consume carbohydrates in every meal.

### 2.3. Oral Glucose Tolerance Test

Blood was collected fasting and 30, 60, 90, and 120 minutes after ingestion of glucose (75 g). Area under the curve of glucose, insulin and C-peptide were calculated [6]. In 3 participants, plasma samples from the OGTT were not stored, hence measurement of insulin and C-peptide are missing for 3 individuals.

### 2.4. Measurement of Muscle, Liver, and Adipose Tissue Insulin Resistance

We performed two-step hyperinsulinemic-euglycemic clamp with deuterated glucose tracers (6,6-^2^H_2_, Cambridge Isotope Laboratories, Tewksbury, MA, USA), as described [6]. The clamp started with 2-h primed (5 mg/kg), continuous (3 mg/kg/h) infusion of [6,6-^2^H_2_] glucose, followed by 2-h infusion of low-dose insulin (15 mU/m^2^/min) and 2-h infusion of high-dose insulin (80 mU/m^2^/min). Deuterated glucose infusion rate was halved (1.5 mg/kg/h) during, and ceased at the end of, the low-dose insulin infusion. Glucose was infused to maintain whole blood concentration of 5.0 mmol/L with variable rate infusion of dextrose (25%, enriched to approximately 2.5% with deuterated glucose). The GIR during the high-dose insulin clamp was calculated at 90–120 min and normalized for fat-free mass (FFM) to produce the M-value.

Deuterated glucose was analyzed by gas chromatography-mass spectrometry (Agilent Technologies, Santa Clara, CA, USA) with correction for natural abundance of ^13^C, as described [16]. EGP was estimated using Steele’s one-compartment fixed-volume model, assuming volume of distribution of 20% of body weight and pool-fraction of 0.65 [17], as modified by Finegood et al. [18]. EGP was fully suppressed during the high-dose insulin infusion, therefore GIR during the high-dose insulin infusion reflects peripheral (mainly muscle) insulin sensitivity. Basal EGP and EGP suppression measure hepatic IR. Adipose tissue IR was evaluated based on the suppression of NEFA in the serum during the low-dose hyperinsulinemic clamp [6]. In two subjects from the original (*n* = 64) cohort, EGP and EGP suppression could not be calculated due to plasma tracer sampling or analysis errors, therefore the metabolomics and lipidomics analyses were performed in 62 individuals.

### 2.5. Body Fat Composition, Abdominal Fat Distribution and Liver Fat

Body fat and FFM were measured using dual-energy X-ray absorptiometry (DXA, Lunar Prodigy, GE-Lunar, WI, USA). Magnetic resonance imaging (MRI, 3.0 T Philips Achieva, Cambridge, MA, USA) images were acquired by mDIXON software (Eindhoven, The Netherland) to assess visceral, subcutaneous, and liver fat. Visceral fat was measured in 5 slices at L4/L5 intervertebral disc level using Image J (1.46r, NIH, Bethesda, MD, USA) and calculated as the difference between total fat and subcutaneous fat. Intra-organ fat from 3 regions of interest in the liver (15 mm × 15 mm) avoiding blood vessels were measured. MRI data were missing for 3 participants who did not fit the MRI scanner [6].

### 2.6. Biochemical Measurements in Blood

Whole blood glucose was measured using the YSI 2300 STAT analyzer (Yellow Springs OH, USA). Insulin and C-peptide were measured by radioimmunoassay (Millipore, St. Charles, MO, USA), lipid profiles by an automated analyzer (Roche, Indianapolis, IN, USA) and NEFA by an enzymatic colorimetric assay (Wako, Osaka, Japan) [6]. Additional fasting whole blood samples were collected into EDTA blood collection tubes, centrifuged at 4 °C, snap frozen on dry ice, and stored at −70 °C until metabolomic and lipidomic analyses.

### 2.7. Targeted Metabolomics

Targeted LC-QQQ-MS analysis was performed to detect a set of water-soluble metabolites in the positive ionization mode using an LC-MS system comprised of an Agilent 1260 Infinity liquid chromatography coupled to a QTRAP 5500 mass spectrometer (AB SCIEX, Framingham, MA, USA). Plasma samples were processed using a method described previously [19]. Briefly, plasma samples were deproteinized using acetonitrile and methanol (75:25; *v*/*v*/*v*) with and without formic acid (0.2%) for analysis on Hydrophilic Interaction Chromatography (HILIC). HILIC-MS-based method, developed for the simultaneous detection of polar metabolites in both positive and negative ionization modes, was used to detect 143 metabolites including, amino acids, nucleotides, high-energy intermediates, organic acids, Krebs cycle intermediates, bile acids, ketones, glycolytic intermediates, neurotransmitters and vitamins, using HILIC columns [19]. Chromatographic columns used for this study and the MS operating conditions are described in the Supplementary material.

### 2.8. Untargeted Lipidomics

Lipidomic profiling was performed on a Q Exactive^TM^ HF-X Hybrid Quadrupole-Orbitrap^TM^ Mass Spectrometer with heated electrospray ionization probe and Thermo Scientific™ Vanquish™ UHPLC system. Plasma for lipidomics were prepared using the modified method described previously [20] with slight modifications described in detail in the Supplementary material. Lipids were quantified by peak area. The estimated concentration of identified lipid species across 14 major lipid classes were calculated relative to the isotopically-labelled internal lipid standards included in the spiked-in SPLASH standard. Lipids with missing values were removed from the analysis, resulting in a lipidomics data set of 775 lipids quantified across all 62 study participants.

### 2.9. Lipid Identification

LipidSearch (v4.1, ThermoFisher, Waltham, MA, USA) mass spectral library was used to identify metabolites out of LC/MS analysis. Over 2300 lipid features were detected in the positive and negative ion mode and the lipids were identified based on accurate mass, MS/MS information, and retention time by matching features in the database. Lipids searched against the standard LipidSearch database comprised three lipid categories: phospholipids, sphingolipids, and neutral lipids. Lipid families identified in this study include ceramide (Cer), sphingomyelin (SM), phosphatidylcholine (PC), lysophosphatidylcholine (LPC), diacylglycerol (DAG), and triacylglycerol (TAG). For each lipid analyte, the first number denotes the total number of carbons in the lipid acyl chain(s), and the number after the colon denotes the total number of double bonds in the lipid acyl chain(s). Due to the similarity of the retention times, lipid isomers including structural (i.e., cis and trans alkenes) and positional isomers (i.e., localization of double-bond position within the fatty acyl chain and stereospecific numbering (sn) of the fatty acyl chain on the glycerol backbone) cannot be distinguished by LC-MS. Isomers of TAGs with the same fatty acyl composition but with small differences in retention times are assigned as “a” and “b”. Low-abundant lipid species such as in the SM and PC classes are presented by the total carbon and double bond number due to inability of the MS/MS to yield characteristic fragments corresponding to two fatty acyl moieties (PC) or by a long-chain base and an *N*-linked acyl moiety (SM).

### 2.10. Data Processing and Analysis

Clinical data were expressed as mean ± SD unless in abnormally distributed data where medians and interquartile ranges (IQR) were provided. Missing data were not imputed.

The Spearman’s rank correlation coefficient, R, and the associated *p*-value between each of the clinical, metabolomic and lipidomic variables was determined. To account for multiple comparisons, the Benjamini–Hochberg procedure was undertaken to determine the significance cutoff for the different comparisons. Briefly, the individual *p*-values were put in ascending order, and the Benjamini–Hochberg critical value calculated, given by the formula (i/m)Q, where i is the individual *p*-value’s rank, m is total number of comparisons, Q is the false discovery rate, taken here to be 0.05. The largest *p*-value that is smaller than the critical value is then taken as the significance cutoff for the correlations.

To explore the clustering of the different variables, a hierarchical cluster tree was created. The data for the clustering were $H=\left(1-p\right)sign\left(R\right)$, where *R* are the values of the Spearman correlations between the variables, for instance between the clinical and the metabolomic variables, *p* is the associated *p*-value and $sign\left(R\right)=\left\{\begin{array}{c} +1,\;R>0\\ \;0\;,\;R=0\\ -1,\;R<0 \end{array}\right.$. The hierarchical cluster tree is determined by calculating the Euclidean distances between the sets of data for each of the variables. The variables were grouped into binary clusters according to distance. These binary clusters are then themselves grouped into larger clusters according to the average distance between the clusters. The clustergram arranges the R-value data firstly according to the hierarchical clustering of the data in the rows and then the hierarchical clustering of the data in the columns. The hierarchical cluster trees are represented by dendrograms in the figures showing the grouping within the clusters. The length of the segments in the dendrograms indicates the average Euclidean distance between the binary pairs. Note that the hierarchical clustering imputes the non-significant R-values with their actual value for the clustergram figures.

To be consistent with insulin resistance, values of M-value, EGP suppression, and NEFA suppression, where a higher value is consistent with less insulin resistance, were multiplied by −1 and named Muscle IR (M-value x−1), Liver IR (EGP suppression x−1), and Adipose IR (NEFA suppression x−1) in the Figures, where relevant.

## 3. Results

### 3.1. Cohort Characteristics

Sixty-two individuals (27 men and 35 women) with an average age of 50.5 (SD 11.4) years were studied. Mean, median, and the ranges of the clinical and metabolic measurements are outlined in Table 1. By design, none of the participants had diabetes. The number of individuals with impaired fasting glucose (IFG), impaired glucose tolerance (IGT) or both IFG and IGT were 1, 11, and 2, respectively. Twenty-one individuals had HbA1c of 39 mmol/mol (5.7%) or over.

### 3.2. Associations between Clinical Variables, Fat Deposition, and Insulin Resistance Phenotypes

**Figure 1:**
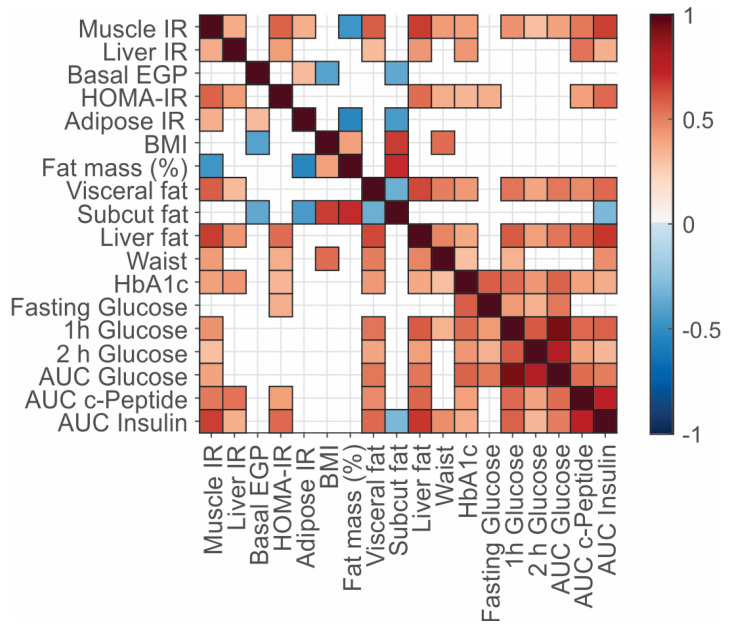
Spearman correlations between the clinical characteristics. The correlations are shown as a heatmap with the color indicating the significance and direction of the correlation (Spearman) (i.e., red positive, blue negative). Only the significant correlations are shown, where the *p*-value cut-off for significance (*p* = 0.025503) was determined using the Benjamini–Hochberg correction. Note the full correlation of the variables with themselves along the diagonal. Complete data were available for all variables other than the abdominal MRI data (*n* = 59) and AUC of insulin and c-peptide (*n* = 57 and *n* = 59, respectively).

Muscle, liver and adipose tissue IR correlated positively with each other. HOMA-IR, considered a measure of hepatic IR, correlated with the gold-standard hepatic IR measure, EGP suppression, but not with basal EGP or adipose tissue IR. Interestingly, HOMA-IR correlated better with muscle IR compared with liver IR (Figure 1). Muscle IR correlated positively with visceral fat, liver fat, HbA1c, and the AUC of insulin, C-peptide, and glucose during the OGTT and inversely with body fat mass (percent of total mass). Adipose tissue IR correlated positively with muscle IR and basal EGP and inversely with subcutaneous fat. Basal EGP correlated inversely with BMI and subcutaneous fat. Fat in the liver correlated more closely with muscle IR than with liver IR and did not correlate significantly with basal EGP. Both visceral fat and liver fat correlated positively and significantly with all the OGTT derived variables, but not with fasting plasma glucose (FPG).

### 3.3. Plasma Omics Signature of Clinical and Metabolic Traits

**Figure 2:**
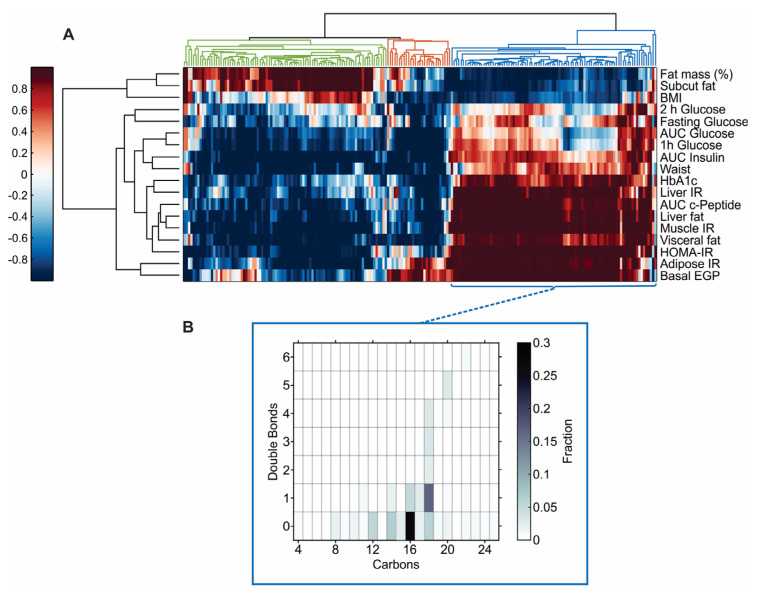
Omics patterns of plasma lipidomics and metabolomics in individuals living with obesity. (**A**) Hierarchical clustering of metabolic phenotypes by plasma lipidomics and metabolomics. (**B**) Chain length (number of carbons) and number of double bonds in the TAGs represented in the blue cluster. In panel A, the clinical variables are shown in the rows with the metabolomics and lipidomics variables in the columns. The *H* = (1 − *p*)*sign*(*R*) value is shown as a heatmap with the color indicating the significance and direction of the correlation (Spearman) (i.e., red positive, blue negative and the darker the color the more significant *p* Value). The variables are clustered firstly according to the clinical variables (rows) with the hierarchical cluster tree shown as a dendrogram on the left. The *p*-value data in the columns (metabolomic and lipidomic variables) were then hierarchically clustered with the dendrogram shown at the top of the heatmap. In panel B, the color indicates the fraction of the total number of fatty acids (*n* = 207 FAs). Complete data were available for all variables other than the abdominal MRI data (*n* = 59) and AUC of insulin and c-peptide (*n* = 57 and *n* = 59, respectively).

We performed hierarchical clustering of the *p*-values between the 18 clinical variables and the omics data. Generally, adiposity (fat mass, subcutaneous fat and BMI, Figure 2A, top, horizontal view), the IR variables (liver IR, muscle IR, adipose IR, basal EGP, and HOMA-IR), liver fat, visceral fat, and the AUC of c-peptide (Figure 2A, bottom, horizontal view) and most of the OGTT-derived variables (Figure 2A, middle, horizontal view) clustered into three distinct groups. the tightest pair of variables, corresponding with the shortest distance of the node from the vertical axis (Figure 2A), was between liver fat and muscle IR.

The lipidomics and metabolomics data clustered into three main groups (vertical view, color coded, Figure 2A). A cluster consisting of 89 nodes (75 lipids and 14 metabolites, left, green) associated positively with adiposity and inversely with most of the other variables. SM and PC species made up most of this cluster (40 and 37%, respectively), while TAGs were not represented. Four ceramide species were included in this cluster, all containing long chain fatty acids (FAs, ≥24 carbons). A small cluster consisting of 28 nodes (24 lipids and 4 metabolites, middle, orange, Figure 2A) correlated positively with adipose tissue IR and basal EGP and inversely with most of the other clinical variables. PC and LPC species made up most of the cluster (orange, 46 and 36%, respectively), while TAGs and SM were not represented. A single ceramide, Cer(d18:2_24:0) was included in this cluster.

Lastly, a cluster of 90 nodes (80 lipids and 10 metabolites, right, blue, Figure 2A) correlated inversely with adiposity and positively with IR phenotypes, C-peptide AUC, liver and visceral fat. 77% of the cluster was made up of TAGs. Most of the TAGs contained saturated and monounsaturated FA with less than 20 carbons (Figure 2B). DAGs made up 4% of this cluster and were not represented in any of the other clusters.

### 3.4. Metabolomics Correlates of Clinical Phenotypes of Obesity

**Figure 3:**
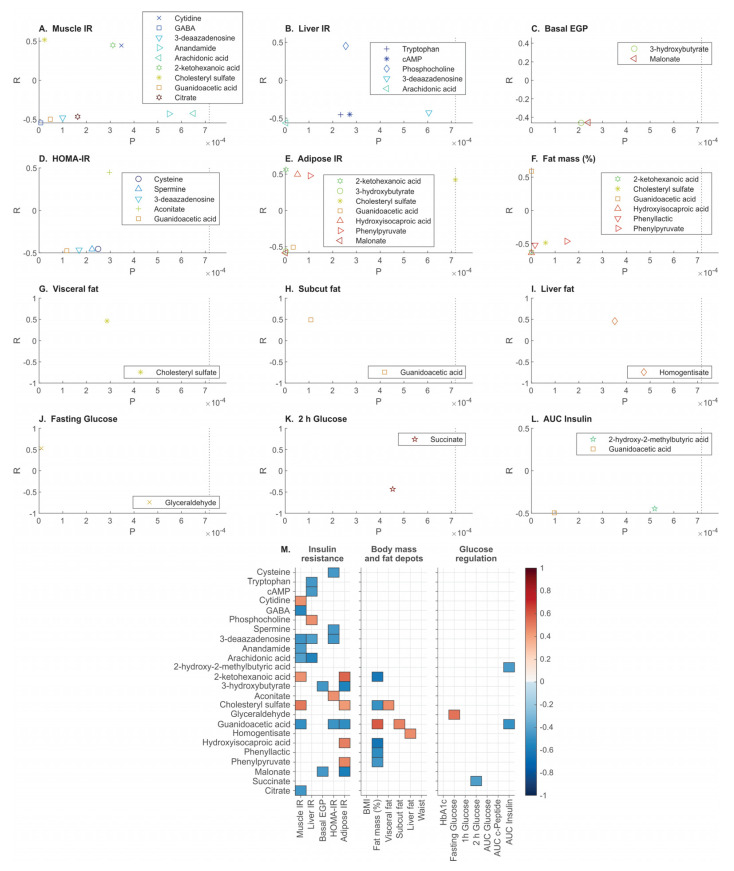
Metabolite correlates of clinical traits in a cohort of people living with obesity (**A**–**L**). Combined Spearman R coefficient for the 25 metabolites which correlated with the different phenotypes. Significant R-values for the Spearman coefficient between metabolites and clinical traits are shown. Only the significant correlations are shown, where the *p*-value cut-off was determined by the Benjamini–Hochberg procedure (*p* = 0.00071671) and depicted in dotted lines (panels **A**–**L**). Non-significant values are not shown. Red- positive and blue- negative R (**M**). Complete data were available for all variables other than the abdominal MRI data (*n* = 59) and AUC of insulin and c-peptide (*n* = 57 and *n* = 59, respectively).

Twenty-five of 143 metabolites correlated significantly with one or more of the phenotypes (Figure 3). Most of the metabolites correlated with more than one IR phenotype (muscle, liver and adipose tissue).

Positive correlations were observed between muscle IR and cholesteryl sulfate, cytidine and 2-ketohexanoic acid, and GABA, guanidinoacetic acid, 3-deaazadenosine, citrate, anandamide (AEA), and arachidonic acid were inversely associated with muscle IR (Figure 3A). Liver IR correlated positively with phosphocholine and inversely with arachidonic acid, tryptophan, cAMP and 3-deaazadenosine (Figure 3B). Malonate and 3-hydroxybutyrate were inversely associated with basal EGP (Figure 3C). HOMA-IR correlated positively with aconitate and inversely with guanidinoacetic acid, 3-deaazadenosine, spermine and cysteine (Figure 3D). Adipose tissue IR correlated positively with 2-ketohexanoic acid, hydroxy-isocaproic acid (HICA), phenylpyruvate and cholesteryl sulfate and inversely with 3-hydroxybutyrate, guanidinoacetic acid and malonate (Figure 3E). Total body fat mass correlated positively with guanidinoacetic acid and inversely with 2-ketohexanoic acid, HICA, phenyllactic acid, cholesteryl sulfate and phenylpyruvate (Figure 3F). Visceral fat correlated positively with cholesteryl sulfate, while subcutaneous fat correlated positively with guanidinoacetic acid (Figure 3G,H). Liver fat correlated positively with homogentisate (Figure 3I). Fasting glucose correlated positively with glyceraldehyde (Figure 3J), and the OGTT derived variables 2-h glucose correlated inversely with succinate (Figure 3K), while the AUC of insulin correlated inversely with 2-hydroxy-2-methylbutyric acid and guanidinoacetic acid (Figure 3L). None of the metabolites correlated significantly with BMI, waist, HbA1c, or with the OGTT variables 1-h glucose, AUC glucose and AUC C-peptide.

Due to their established positive association with whole body IR [21], we examined the relationships between the plasma branched-chain amino acids (BCAAs; valine, leucine and isoleucine) and the IR sites. We found positive correlations specifically with muscle IR (r = 0.25–0.29, 0.01 < *p* < 0.05), but the associations did not withstand the Benjamini–Hochberg statistical correction (*p* ≤ 0.0007).

Amongst the clamp measured IR variables, four (cytidine, GABA, anandamide, and citrate), three (tryptophan, cAMP and phosphocholine), and two (phenylpyruvate and HICA) metabolites corresponded uniquely with muscle-, liver-, or adipose tissue-IR, respectively (Figure 3M). Out of the fat depots, visceral fat correlated uniquely with cholesteryl sulfate, while subcutaneous fat correlated uniquely with guanidinoacetic acid, and liver fat correlated uniquely with homogentisate (Figure 3M).

## 4. Discussion

Insulin resistance measured in muscle, liver and adipose tissue clustered together and were indistinguishable by plasma ‘omics signatures in a cohort of sixty-two individuals living with obesity. However, few metabolites correlated exclusively with muscle-IR (cytidine, GABA, anandamide and citrate), liver-IR (tryptophan, cAMP and phosphocholine), and adipose tissue-IR (phenylpyruvate and HICA); cholesteryl sulphate and guanidinoacetic acid differentiated between adverse and benign adiposity.

Several lines of evidence demonstrate that the composition of specific fatty acids in TAGs explains the relationship between plasma TAGs and insulin resistance or type 2 diabetes [14,22]. In our study, TAGs with shorter chain fatty acids (12–18 carbons) and higher degree of saturation (double bond content ≤1) were elevated in the settings of insulin resistance in skeletal muscle, liver and adipose tissue. Specifically, lauric (12:0), myristic (14:0), palmitic (16:0), palmitoleic (16:1), stearic (18:0), and oleic (18:1) acids as constituents of TAGs were significantly elevated in the context of IR. Our findings are consistent with previous studies [14,22] and provide a higher level of resolution into the tissues involved in insulin resistance.

### 4.1. Biomarkers Aligning with Muscle Insulin Resistance

Skeletal muscle is the largest glucose storage depot in the body, storing approximately 80% of total body glycogen. After ingestion of carbohydrates, the muscle is responsible for 30–40% of glucose uptake [23]. Under normal conditions, when faced with high glucose, the ability of the muscle to dispose glucose reduces the insulin secretory burden on the beta cells [24]. However, the engagement of the muscle in glucose disposal is impaired with long-term exposure to an obesogenic environment associated with the development of type 2 diabetes. We found that circulating cytidine associated strongly with muscle IR, in line with previous findings in type 2 diabetes [25]. Our metabolomics analysis indicated inverse associations of muscle IR with GABA, a neurotransmitter synthesized from glutamate by glutamic acid decarboxylase. GABA was recently shown to exert antidiabetic effects by acting on beta cells [26].

Interestingly, we found that circulating citrate was inversely correlated with muscle IR. Although mitochondria are a major source of citrate production for most mammalian cells, plasma contains relatively high concentrations of citrate [27]. Cellular citrate plays significant roles in regulating glycolysis and gluconeogenesis rates in tissues; high levels suppress glycolysis and stimulate gluconeogenesis, and vice versa. It is unclear if the inverse association with muscle IR reflects increased uptake of extracellular citrate to maintain cell energy homeostasis.

Skeletal muscle response to insulin is regulated by several factors including growth hormone, cytokines secreted by inflammatory cells and adipocytes, and FAs and their derivatives, including the endocannabinoids [28]. We found that AEA, a lipid neurotransmitter derived from arachidonic acid, was inversely associated with muscle IR. AEA has been suggested to play a role in regulating appetite and energy expenditure [29] and plasma AEA was elevated in obesity and suppressed with insulin infusion [30], consistent with our findings.

### 4.2. Biomarkers Aligning with Liver Insulin Resistance

After a meal containing carbohydrates, the liver not only disposes approximately one third of the glucose, but it also suppresses glucose production and release, together estimated to be responsible for approximately 60–65% of the disposed glucose load [23]. In healthy individuals, EGP was suppressed rapidly in response to glucose ingestion, regardless of the glucose dose (25, 50, and 75 g) [24] and hypercaloric dietary interventions resulted in impairment in EGP suppression within days in healthy individuals [31].

Associations of aromatic amino acids with diabetes risk have been observed in various populations [32,33]. Dysregulation in tryptophan (Trp) metabolism has been shown to be highly associated with IR and diabetes risk [34]. A recent study demonstrated that circulating Trp was significantly associated with a decrease in insulin secretion, but not with IR [35]. We found that liver IR was negatively correlated with plasma Trp. cAMP is a potent amplifier of the insulin secretory response to glucose and is involved in the regulation of the hepatic enzyme Trp-2,3-dioxygenase, the first and rate-limiting enzyme in the kynurenine pathway of Trp metabolism [36]. Consistent with the inverse association between liver IR and Trp, cAMP followed the same direction in our study. In contrast to our findings, a previous study reported positive correlations between obesity and type 2 diabetes with plasma cAMP [37], but the specificity with liver IR has not been reported.

Phosphatidylcholines (PC), an important phospholipid component of cellular membranes including of plasma lipoproteins, were inversely correlated with IR in both muscle and liver. Similarly, lipidomic analysis of skeletal muscle biopsies from human donors demonstrated a negative correlation between total PC and IR measured by frequently sampled intravenous glucose tolerance test in the donors [38]. DAGs, on the other hand, are key lipid intermediates proposed to mediate IR. Our findings in plasma agree with previous studies which implicated the association between IR and accumulation of DAGs in the liver [39]. Interestingly, we found a strong positive association between circulating phosphocholine (p-choline, headgroup of PC) with liver IR, suggesting enhancement of phospholipase C activity, as has been implicated in diabetes [40].

### 4.3. Biomarkers Aligning with Adipose Tissue Insulin Resistance

Circulating HICA, an end-product of leucine metabolism in human tissues, correlated uniquely and strongly with adipose tissue IR. Adipose tissue plays essential roles in maintaining lipid and glucose homeostasis and is an important regulator of BCAAs metabolism and their biotransformation to lipids for storage. Although we did not observe significant associations between leucine and adipose tissue IR, the findings in relation to HICA are in line with previous reports linking adipose tissue with BCAA metabolism [41,42].

Similarly, we found positive association between circulating phenylpyruvate, an intermediate in the catabolism of the aromatic amino acid phenylalanine, but not phenylalanine itself, with adipose tissue IR. Perturbation of phenylalanine and tyrosine metabolism in IR states have been shown to precede alteration in BCAA metabolism [43], and circulating aromatic amino acids such as phenylalanine and tyrosine have been consistently associated with risk of type 2 diabetes [44].

### 4.4. Biomarkers Aligning with Abdominal Fat Depots

Visceral fat is closely associated with insulin resistance, prediabetes and type 2 diabetes, while the abdominal subcutaneous fat stores are not considered metabolically adverse [45]. In this cohort of people living with obesity we found that not only BMI and fat mass, but also abdominal subcutaneous fat were benign markers of adiposity, while visceral fat and liver fat were confirmed metabolically adverse.

We found that visceral fat, but not subcutaneous fat was associated positively with plasma cholesteryl sulfate. Cholesteryl sulfate is transported in part via LDL and was previously reported to be significantly elevated in liver cirrhosis and hypercholesterolemia [46]. Together with positive associations with muscle IR and adipose tissue IR in our study, we suggest that circulating cholesteryl sulfate may serve as a plasma biomarker of metabolic risk. On the other hand, plasma guanidinoacetic acid may serve as a measure of benign adiposity. Guanidinoacetic acid is a precursor of creatine and a key player in maintaining cellular bioenergetics [47]. Plasma guanidinoacetic acid appeared to be strongly correlated with fat mass and subcutaneous fat, consistent with a previous study [48] and inversely associated with muscle IR, HOMA-IR, adipose tissue IR and the insulin AUC during the OGTT.

A central intermediate of phenylalanine and tyrosine catabolism, homogentisate, correlated uniquely and positively with liver fat in our study. While plasma tyrosine was previously reported to correlate with liver fibrosis stage in individuals with nonalcoholic steatohepatitis [49], homogentisate has not been previously reported to relate to metabolic disease. Our findings suggest that plasma homogentisate may serve as biomarker of liver fat content in people living with obesity.

### 4.5. Study Limitations

The relatively small cohort was dictated by the application of detailed methodologies to comprehensively phenotype the study participants. Furthermore, plasma levels of metabolites do not directly reflect cellular metabolism. However, previous studies suggested that circulating metabolites are highly sensitive to altered cellular metabolic processes [50]. Finally, the observational nature of the study precludes conclusions about cause and effect and the findings may not be generalized to individuals with overt diabetes.

In summary, we report plasma metabolites which uniquely aligned with insulin resistance phenotypes and abdominal fat depots in obesity. Our findings may have clinical implications; however, the utility of the biomarkers identified here should be validated.

## Figures and Tables

**Table 1:** Clinical and metabolic characteristics of the cohort.

Class of Measurement	Characteristic	Mean/Median (± SD or IQR)	Min-Max Values
	N (M/F)	62 (27/35)	
	Age (years)	50.5 ± 11.4	21–69
Body mass, fat composition and distribution	BMI (kg.m^−2^)	36.3 ± 4.5	30.9–48.5
	Waist circumference (cm)	111 ± 14	85–155
	Body fat (% of total mass)	48 (40, 52)	31–61
	Visceral fat (cm^3^)	249 (212, 320)	139–492
	Subcutaneous fat (cm^2^)	501 (418, 596)	265–835
	Liver fat (%)	10.6 (5.9, 21.3)	0.7–63.5
Blood pressure	Systolic (mm Hg)	125 ± 13	100–169
	Diastolic (mm Hg)	82 ± 9	59–106
Serum lipids	Total cholesterol (mmol.L^−1^)	4.9 ± 0.8	3.1–7.1
	LDL cholesterol (mmol.L^−1^)	3.0 ± 0.7	1.5–5.0
	HDL cholesterol (mmol.L^−1^)	1.3 ± 0.3	0.8–2.0
	Triglycerides (mmol.L^−1^)	1.1 ± 0.4	0.4–2.5
	NEFA (mmol.L^−1^)	0.4 ± 0.1	0.1–0.7
Glucose regulation	HbA1c (mmol/mol)	36 ± 3	29–43
	HbA1c (%)	5.5 ± 0.3	4.8–6.1
	Fasting blood glucose (mmol.L^−1^)	4.8 ± 0.4	3.5–6.0
	2-hour blood glucose (mmol.L^−1^)	6.3 ± 1.6	2.7–10.1
	Individuals with IFG (*n*)	1	
	Individuals with IGT (*n*)	12	
	Individuals with IFG and IGT (*n*)	2	
	Individuals with HbA1c ≥ 5.7%	21	
	Fasting insulin (mU.L^−1^)	16.5 (11.3, 27.4)	5.9–73.4
	HOMA-IR	3.4 (2.2, 6.2)	1.2–19.6
	EGP (mg.kg^−1^.min^−1^)	2.1 ± 0.3	1.4–2.8
	EGP suppression (%)	65 ± 14	30–93
	GIR/FFM (M-value, µmol.min^−1^.kg^−1^)	90 ± 30	24–186
	NEFA suppression (%)	32 ± 13	10–62
Serum insulin during the clamp (mU.L^−1^)	Low dose clamp	42 ± 13	19–86
	High dose clamp	212 ± 44	131–296
Medication use	Individuals treated with anti-hypertensive medications (*n* (%))	13 (21)	
	Individuals treated with lipid reducing agents (*n* (%))	8 (13)	

Table footnotes: Values are mean ± SD or median (25th, 75th percentile) and minimal to maximal. Complete data were available for all variables other than the abdominal MRI data (*n* = 59; variables affected: visceral fat, subcutaneous fat and liver fat). Impaired fasting glucose (IFG) 5.6–6.9 mmol/L; impaired glucose tolerance (IGT) 2-h plasma glucose during OGTT (75-g glucose) 7.8–11.0 mmol/L.

## Data Availability

The data presented in this study are available on request from the corresponding author. The participants have consented to research data gathered from the results of the study to be published. The data are not publicly available because the participant information and consent form did not include sharing of data.

## Supplementary Materials

The following supporting information can be downloaded at: https://www.mdpi.com/article/10.3390/metabo12121272/s1. Further details on the targeted metabolomics and on the lipidomics. Refs [51,52] are mentioned in the file.
