# Supplementary material for: Metabolomics and Lipidomics Signatures of Insulin Resistance and Abdominal Fat Depots in People Living with Obesity
Source: Metabolites. 2022 Dec 15;12(12):1272. doi: 10.3390/metabo12121272 (PMC9781703; doi:10.3390/metabo12121272)
Supplement: Supplementary file 1 [file metabolites-12-01272-s001.zip › metabolites-2085667-supplementary.pdf]

Supplementary material

Targeted metabolomics

Chromatographic columns used for this study and the MS operating conditions

A Waters XBridge BEH Amide column (2.1 × 100 mm, 3.5 µm; Waters; Milford, MA) was used in the negative ionization mode with mobile phase (A) consisted of 20 mM ammonium acetate and 20 mM ammonium hydroxide in water:acetonitrile (95:5 v/v) at pH 9; mobile phase (B) consisted of acetonitrile (ACN). A Waters Atlantis HILIC Silica column (2.1 × 150 mm, 3 µm; Waters; Milford, MA) was used in the positive ionization mode; mobile phase (A) consisted of water containing 0.1% formic acid and 10 mM ammonium formate; mobile phase (B) consisted of acetonitrile with 0.1% formic acid. The LC was connected to an AB Sciex Triple Quad 5500 mass spectrometer run in positive ion mode, on which the MRM conditions of 165 polar metabolites were optimized for maximum sensitivity by direct infusion of individual standard solutions at concentration ranging from 100 ng/mL to 1000 ng/mL. The LC was connected to an AB Sciex Triple Quad 5500 mass spectrometer operated in both ionization modes, on which the MRM conditions of 164 polar metabolites were optimized for maximum sensitivity by direct infusion.

Ionization was achieved using electrospray ionization (ESI) in the positive and negative modes at 350 °C with N_2 as nebulizer. The MS source parameters were set at: source temperature: 350 °C, high ionization voltage: 5500 V, curtain and nebulizer gases: nitrogen; 25, 30, and 30 psi; respectively. MS/MS acquisition was realized in scheduled reaction monitoring mode with optimal collision energy, collision cell exit potential and declustering potential values using metabolite standard solutions. Quality control (QC) pooled plasma samples were prepared by mixing 20 µL of each individual sample of the study and were included in the analytical run, spaced at regular intervals of every 10 injections, enabling the monitoring and correction for temporal drift in mass spectrometry performance. The nearest-neighbor flanking pair of pooled plasma was used to normalize experimental samples in a metabolite-by-metabolite manner. 143 metabolites from the 165 metabolites optimized for both positive and negative modes detection were detectable in the plasma extracts. Raw data files (Analyst software, version 1.6.2; AB Sciex, Foster City, CA, USA) were imported into the provided analysis software Multi-Quant 3.0 for MRM Q1/Q3 peak integration and data were normalized relative to pooled plasma samples that were analyzed in the sample queue after every 10 study samples.

Lipidomics

Lipid extraction method

Thirty (30) µL of plasma was spiked with 10 µL of SPLASH standard before lipid extraction and were extracted using methyl tert-butyl ether (MTBE): methanol: water (10:3:2.5), dried down and reconstituted into 100 µL of IPA/MeOH (1:1) giving a 1:10 dilution of the internal standards. Samples were resolved on a 2.1 × 150 mm Thermo Scientific™ Acclaim™ C30 column (3 µm pore size), using a binary gradient in which mobile phase A consisted of acetonitrile: water (60:40) with 10 mM ammonium formate and 0.1% formic acid; and mobile phase B was isopropanol: acetonitrile (90:10) with 10 mM ammonium formate and 0.1% formate acid. The chromatographic separation was carried out at a flow rate of 260 µL min^{-1} with 5 µL injection volume using the following gradient: 0–2 min ramp from 30% to 55% B, 2-12 min ramp to 65% B, 12-18 min ramp to 85% B, 18-20 min ramp to 100% B, before back to initial mobile phase conditions of 30% B, and the column was held for 6 min at 30% B until the next sample injection. The injector needle was washed with 75% IPA, 24.9% H2O, and 0.1% formic acid prior to each injection. The column oven temperature was set to 50 °C. Data were acquired in full scan with data-dependent MS^2 ($ddMS^2$) acquisition (full scan at resolution of 120k FWHM and the scan range of 250-1200 amu in positive ionization mode; and 250-1000 amu in negative ionization mode), as described previously [51,52]. The 20 most abundant ions in each cycle were subjected to MS^2 at the resolution of 17,500 FWHM. Isolation window was set to 1.4 m/z,

collision energy to 20, 30, 60 eV, maximum integration time 110 ms and dynamic exclusion window 10 s. An exclusion list of background ions was used based on a solvent blank. LipidSearch v4.1.30 software (ThermoFisher Scientific) was used for chromatogram alignment, peak identification and integration. Each lipid peak was manually verified for column elution time and characteristic fragment ions. Internal standard peak areas were monitored for quality control and used to normalize analyte peak areas. In addition, we performed lipid profiling on pooled plasma samples, generated from a distinct 10 μl aliquot of pooled plasma, extracted, and processed individual and spaced every 10 experimental samples in the overall sample queue, enabling detection of temporal drift in instrument performance.

References

51. Kiyonami, R.; Peake, D.A.; Liu, X.; Huang, Y. Large-scale lipid profiling of a human serum lipidome using a high-resolution, accurate-mass LC/MS/MS approach. In Proceedings of the LIPID MAPS Annual Meeting, La Jolla, CA, USA, 17–18 May 2016; pp. 12–13.
52. Peake, D.A.; Kiyonami, R.; Yokoi, Y.; Fukamachi, Y.; Huang, Y. Processing of a complex lipid dataset for the NIST inter-laboratory comparison exercise for lipidomics measurements in human serum and plasma. In Proceedings of the ASMS Annual Meeting, Saint Louis, MO, USA, 31st May–4th June 2015.
